# High Magnesium and Sirolimus on Rabbit Vascular Cells—An In Vitro Proof of Concept

**DOI:** 10.3390/ma14081970

**Published:** 2021-04-14

**Authors:** Giorgia Fedele, Sara Castiglioni, Jeanette A. Maier, Laura Locatelli

**Affiliations:** 1 Department of Biomedical and Clinical Sciences L. Sacco, Università di Milano, Via GB Grassi 74, 20157 Milano, Italy; giorgia.fedele@unimi.it (G.F.); sara.castiglioni@unimi.it (S.C.); laura.locatelli@unimi.it (L.L.); 2 Interdisciplinary Centre for Nanostructured Materials and Interfaces (CIMaINa), Università di Milano, 20133 Milan, Italy

**Keywords:** magnesium, sirolimus, rabbit coronary artery endothelial cells, smooth muscle cells

## Abstract

Drug-eluting bioresorbable scaffolds represent the last frontier in the field of angioplasty and stenting to treat coronary artery disease, one of the leading causes of morbidity and mortality worldwide. In particular, sirolimus-eluting magnesium-based scaffolds were recently introduced in clinical practice. Magnesium alloys are biocompatible and dissolve in body fluids, thus determining high concentrations of magnesium in the local microenvironment. Since magnesium regulates cell growth, we asked whether high levels of magnesium might interfere with the antiproliferative action of sirolimus. We performed in vitro experiments on rabbit coronary artery endothelial and smooth muscle cells (rCAEC and rSMC, respectively). The cells were treated with sirolimus in the presence of different concentrations of extracellular magnesium. Sirolimus inhibits rCAEC proliferation only in physiological concentrations of magnesium, while high concentrations prevent this effect. On the contrary, high extracellular magnesium does not rescue rSMC growth arrest by sirolimus and accentuates the inhibitory effect of the drug on cell migration. Importantly, sirolimus and magnesium do not impair rSMC response to nitric oxide. If translated into a clinical setting, these results suggest that, in the presence of sirolimus, local increases of magnesium concentration maintain normal endothelial proliferative capacity and function without affecting rSMC growth inhibition and response to vasodilators.

## 1. Introduction

Coronary artery disease exacts a very high toll in terms of morbidity and mortality all over the world [1]. While prevention and lifestyle changes remain the best weapons to control the disease, it might be necessary to resort to invasive procedures that increase blood supply to the heart. Since the implantation of the first bare metal stents in 1986, innovative solutions have been adopted to limit in-stent restenosis, stent thrombosis and, as recently reported, neoatherosclerosis [2]. Antiproliferative drug eluting stents were generated and percutaneous coronary intervention involving these devices is now widely utilized to treat artery stenosis and prevent re-stenosis [3,4]. The last frontier for coronary stenting was the development of bioresorbable scaffolds (BRS), which provide transient vessel support and are gradually dissolved to avoid the permanent caging of the vessel, thus granting the restoration of vasomotion [5]. In this field, magnesium (Mg) alloys raised major interest because of their mechanical and biological features [6]. Indeed, they possess an adequate radial force and are biocompatible, since magnesium is an essential element involved in myriads of physiological processes [7]. In addition, Mg scaffolds are resorbed by the body with no adverse effects. Moreover, Mg is beneficial for the arteries and, in particular, it positively impacts endothelial function by preventing oxidative stress and inhibiting pro-inflammatory events [8,9,10]. Sirolimus eluting biodegradable Mg scaffolds have recently been introduced into clinical practice, with favorable outcomes [11].

Sirolimus (also known as rapamycin) is a macrolide with potent immunosuppressive and anti-proliferative properties because it inhibits the mammalian target of rapamycin (mTOR), a highly conserved serine/threonine kinase that regulates crucial cell functions such as metabolism, growth, survival, autophagy and transcription [12]. Sirolimus is used for coronary stent coating, since it brakes the proliferation of medial smooth muscle cells, reducing in-stent restenosis [13]. However, vascular dysfunction has been reported in rats after continuous sirolimus infusion at a rate of 5 mg/kg/day, reaching a blood concentration as high as 14.2 ± 5.7 µg/L [14]. Similarly, in isolated human thoracic arteries, sirolimus impaired endothelial function [15]. In cultured human umbilical vein endothelial cells, a widely used in vitro model of macrovascular endothelial cells, conflicting results are available. A first study demonstrated an impairment of cell viability and function with concentrations of sirolimus higher than 1 nM [16]. In the same cells, another group reported the beneficial effect of sirolimus (100 ng/mL) in inhibiting TNFα-induced VCAM upregulation, thus reducing the recruitment of leukocytes into inflamed sites [17].

Studies from preclinical models implanted with Mg-based sirolimus eluting scaffolds yielded promising results. Mg-based BRS are safe, induce healing and show low thrombogenicity in rabbits and pigs [2]. These scaffolds also reduce neo-atherosclerosis in rabbits [2].

The purpose of the present in vitro study was to assess the effects of different concentrations of sirolimus and Mg in rabbit coronary artery endothelial and smooth muscle cells (rCAEC and rSMC, respectively). Since Mg stimulates cell growth and prevents apoptosis, we wondered whether an increase in extracellular Mg concentration might reduce the anti-proliferative effects of sirolimus on rSMC and counterbalance possible detrimental effects of sirolimus in rCAEC. A two-fold increase in Mg concentration was measured in the wall of pig coronary arteries, after the implantation of a Mg-alloy scaffold [18]. Therefore, we cultured the cells in medium containing from 1, which is the physiological concentration, to 3 mM Mg. To our knowledge, no data are available about the concentration of sirolimus in the vessel wall after scaffold implantation. Studies in the literature indicate that sirolimus is about 1.5 µg/mm^2^ of scaffold surface and it degrades in about 90 days [19]. We decided to utilize 2 to 50 ng/mL of sirolimus.

## 2. Materials and Methods

### 2.1. Cell Culture

Primary rCAEC and rSMC were purchased from Creative Bioarray (Shirley, NY, USA) and expanded for 3 population doublings, according to the manufacturer’s instructions. To increase Mg concentration from 1 to 1.5, 2 and 3 mM Mg, MgSO_4_ was added to the media. The cells were also treated with different concentrations of sirolimus: 2 ng/mL, 10 ng/mL or 50 ng/mL. The experiments were conducted for 24, 48, 72 and/or 144 h. Different batches of cells were utilized to overcome possible differences due to individual variability. The cells were trypsinized and stained with trypan blue solution (0.4%). Viable cells were counted using a Luna Automated Cell Counter (Logos Biosystems, Anyang, Korea). All the experiments were performed at least three times.

### 2.2. In Vitro Wound Assay

Primary rCAEC and rSMC were seeded on 24-well plates (Greiner bio-one, Frickenhausen, Germany) and cultured in the presence of 1 or 3 mM of extracellular Mg with or without sirolimus (2 or 50 ng/mL). After 48 h of treatment, the monolayers were scraped, washed with PBS twice to remove debris and maintained in media containing Mg and/or sirolimus for an additional 24 h. Then, the cells were fixed and colored with Crystal Violet for 20 min, washed with PBS twice and visualized by optical microscope (4X magnification). The percentage of migration vs. CTR (1 mM Mg with no sirolimus) was analyzed using ImageJ [20].

### 2.3. Fluorescence Microscopy

The cells were grown on microscope glasses, fixed in PBS containing 3% paraformaldehyde and 2% sucrose (pH 7.6), and stained with fluorescent phalloidin to visualize the cytoskeleton or with 4′,6-diamidino-2-phenylindole (DAPI) to detect the nuclei. Finally, cells were mounted with moviol, and images were acquired using a 63X objective in oil by an SP8 confocal microscope (Leica Microsystems, Buffalo Grove, IL, USA). Magnification was 40X.

### 2.4. Enzyme-Linked Immunosorbent Assay–ELISA

The cells were seeded on 24-well plates and treated for 72 and 144 h in the presence of 1 or 3 mM of extracellular Mg with or without sirolimus (2 or 50 ng/mL). For the quantitative determination of rabbit tissue factor (tF), P-selectin, Vascular Cell Adhesion Protein 1 (VCAM) (Cloud-clone corp., Katy, TX, USA) and endothelial Nitric Oxide Synthase (eNOS) (Creative Diagnostics, New York, NY, USA) ELISA kits were used according to the datasheet instructions. All the ELISA were performed on 30 µg of cell extracts in triplicates for 3 times.

### 2.5. Cyclic GMP Measurement

rSMC were seeded on 24-well plates and cultured for 72 h in the presence of 1 or 3 mM Mg, combined or not with sirolimus (2 and 50 ng/mL). The cells were then treated for 30 min with Sodium Nitroprusside (SNP 10 µM) (Sigma Aldrich, St. Louis, MO, USA), a donor of nitric oxide (NO). The Cyclic GMP XP Assay Kit (Cell Signaling, Danvers, MA, USA) was utilized according to the manufacturer’s instructions. The percentage of activity was calculated as follows: % activity = 100 × [(A − Abasal)/(Amax − Abasal)], where A is the sample absorbance, Amax is the absorbance at maximum stimulation (high cyclic GMP concentration), and Abasal is the absorbance at basal level (no cGMP). The experiment was performed 3 times in triplicate.

### 2.6. Statistical Analysis

Data are reported as means ± SD. The data were normally distributed and they were analyzed using one-way repeated measures ANOVA. The p-values deriving from multiple pairwise comparisons were corrected by the Bonferroni method. Statistical significance was defined for *p*-value ≤ 0.05. * *p* ≤ 0.05; ** *p* ≤ 0.01; *** *p* ≤ 0.001; **** *p* ≤ 0.0001.

## 3. Results

### 3.1. The Effect of Sirolimus and Mg on rCAEC Growth, Migration and Morphology

**Figure 1:**
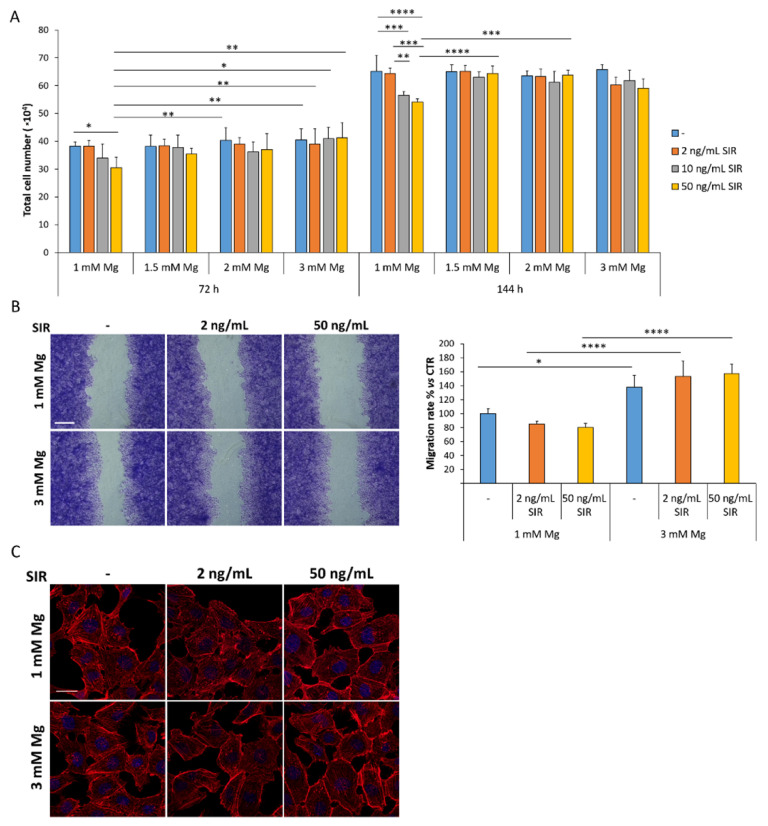
The effects of sirolimus and Mg on rCAEC. (**A**) rCAEC were counted after 72 and 144 h of culture in medium containing 1, 1.5, 2 or 3 mM Mg with or without different concentrations of sirolimus (SIR, 2, 10, 50 ng/mL). -SIR refers to untreated controls. (**B**) Confluent rCAEC were cultured in the presence of 1 or 3 mM Mg combined with 2 or 50 ng/mL of sirolimus for 48 h. Then wound assay was performed. 24 h later the width of the wound was visualized and measured. Representative images of the scratches are shown (left panel). Scale bar: 125 μm. The migration rate was analyzed using ImageJ and data are shown as the percentage of migration compared to the control (CTR, 1 mM Mg without sirolimus, right panel). (**C**) Confluent rCAEC were cultured in culture medium containing 1 or 3 mM Mg with or without sirolimus (2 and 50 ng/mL), stained with phalloidin and DAPI and visualized by fluorescence microscopy. Representative images are shown. Scale bar: 20 μm. Data are expressed as the mean ± standard deviation of three independent assays (* *p* ≤ 0.05; ** *p* ≤ 0.01; *** *p* ≤ 0.001; **** *p* ≤ 0.0001).

rCAEC were seeded at low density and cultured in the presence of 2, 10 and 50 ng/mL of sirolimus in media containing different concentrations of Mg (1 to 3 mM). After 72 or 144 h the cells were trypsinized and counted. Figure 1A shows that sirolimus inhibits rCAEC proliferation only in 1 mM Mg, while concentrations higher than 1 mM Mg prevent this inhibitory effect.

We then performed all the following experiments utilizing media containing 1 or 3 mM, with or without 2 and 50 ng/mL of sirolimus. We also found that culture for 72 h in 3 mM Mg with or without sirolimus enhances rCAEC migration (Figure 1B). Moreover, no significant differences were detected in cell morphology, nuclei and cytoskeleton in rCAEC treated with 2 and 50 ng/mL of sirolimus in medium containing 1 or 3 mM Mg for 72 h, after staining with fluorescent phalloidin and DAPI to visualize the cytoskeleton and the nuclei (Figure 1C).

### 3.2. The Effect of Sirolimus and Mg on the Amounts of VCAM and P-Selectin in rCAEC

**Figure 2:**
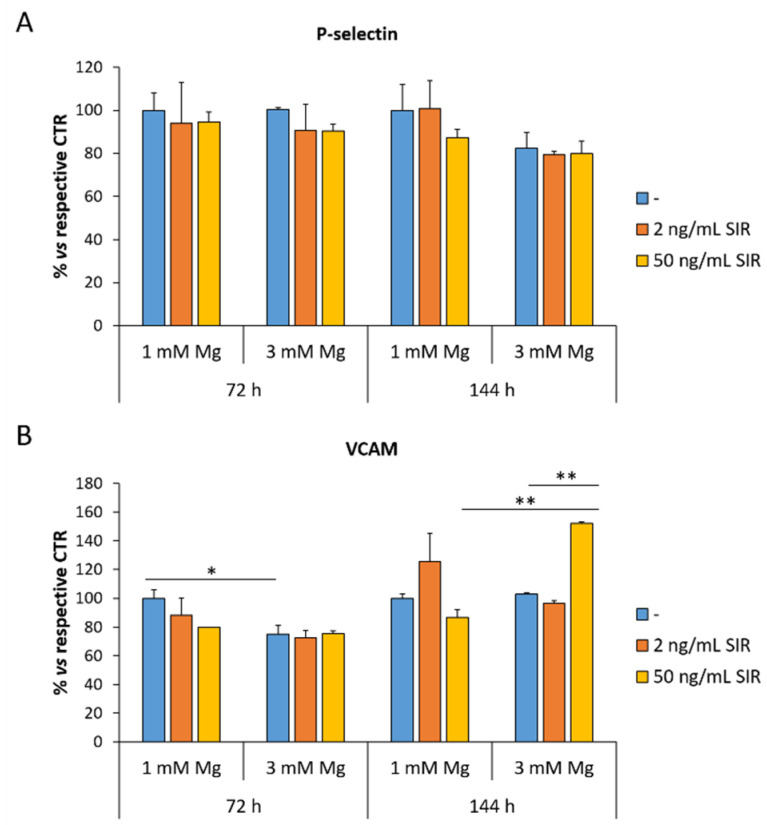
The effects of sirolimus and Mg on the total amounts of P-selectin and VCAM. ELISA was performed on cell extracts after 72 and 144 h of culture in medium containing 1 or 3 mM Mg in the presence of 2 or 50 ng/mL of sirolimus. (**A**) P-selectin (**B**) VCAM. Data are expressed as the mean ± standard deviation of three independent assays (* *p* ≤ 0.05; ** *p* ≤ 0.01).

When activated or dysfunctional, endothelial cells upregulate adhesion molecules to recruit leukocytes [16]. The cells were treated with 2 or 50 ng/mL of sirolimus in media containing 1 or 3 mM Mg for 72 and 144 h. By ELISA, we measured the amounts of VCAM and P-selectin, both involved in atherogenesis. While P-selectin is not modulated by extracellular Mg and sirolimus (Figure 2A), VCAM is downregulated in 3 mM Mg also in the presence of sirolimus at 72 h and upregulated in rCAEC cultured in 3 mM Mg with 50 ng/mL sirolimus for 144 h (Figure 2B). 

### 3.3. The Effect of Sirolimus and Mg on the Amounts of tF and eNOS in rCAEC

**Figure 3:**
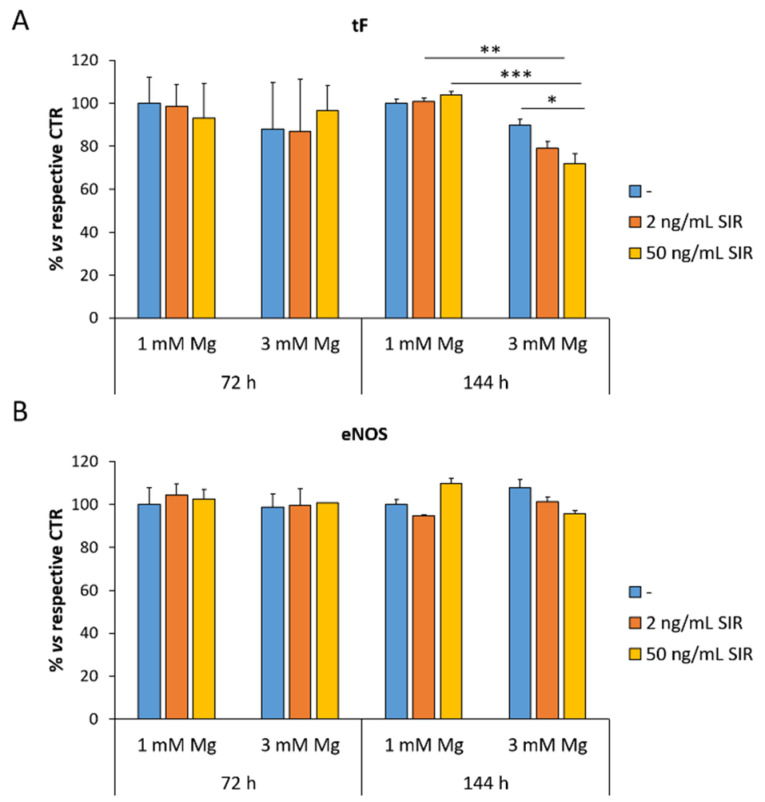
The effects of sirolimus and Mg on the total amounts of tF and eNOS. ELISA for tF (**A**) and eNOS (**B**) was performed as described above. Data are expressed as the mean ± standard deviation of three independent assays (* *p* ≤ 0.05; ** *p* ≤ 0.01; *** *p* ≤ 0.001).

Dysfunctional endothelial cells upregulate tF, which activates the extrinsic coagulation pathway and triggers thrombosis [21]. The total amounts of tF were measured by ELISA on rCAEC cultured as described above. Figure 3A shows no modulation of tF after 72 h of culture in the presence of sirolimus or 3 mM Mg alone, while the total amount of tF is significantly reduced in cells cultured in 3 mM Mg with sirolimus for 144 h. Moreover, since aberrant levels of eNOS have been described in atherosclerosis [22], we analyzed the levels of the enzyme in rCAEC under the same experimental conditions and did not detect significant differences (Figure 3B).

### 3.4. The Effect of Sirolimus and Mg on rSMC Growth and Migration

**Figure 4:**
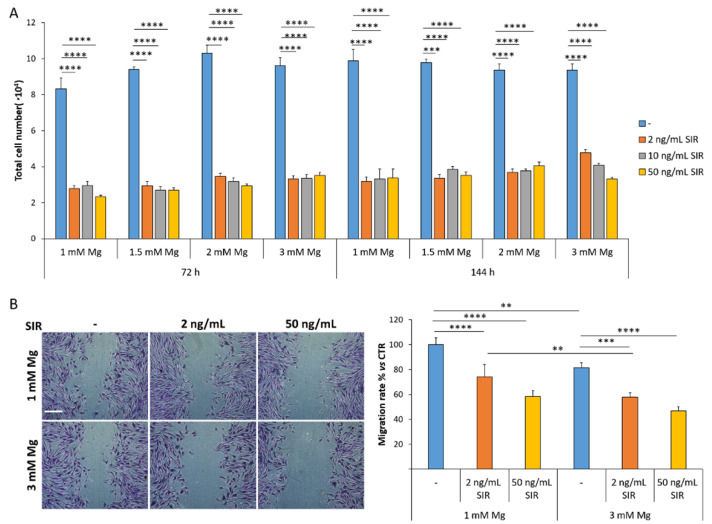
The effects of sirolimus and Mg on rSMC. (**A**) The cells were counted after 72 and 144 h of culture in medium containing 1, 1.5, 2 or 3 mM Mg with or without different concentrations of sirolimus (2, 10, 50 ng/mL). (**B**) Confluent rSMC were cultured in the presence of 1 or 3 mM Mg combined with 2 or 50 ng/mL of sirolimus for 72 h and the wound assay was performed. 24 h later, the width of the wound was visualized and measured. Representative images of the scratches are shown (left panel). Scale bar: 125 μm. The migration rate was analyzed using ImageJ and data are shown as the percentage of migration compared to the control (CTR, 1 mM Mg without sirolimus, right panel). Data are expressed as the mean ± standard deviation of three independent assays (* *p* ≤ 0.05; ** *p* ≤ 0.01; *** *p* ≤ 0.001; **** *p* ≤ 0.0001).

rSMC were cultured with or without 2, 10, 50 ng/mL sirolimus in the presence of different concentrations of extracellular Mg, from 1 to 3 mM, for 72 and 144 h. After 72 h control cells were confluent, while a marked growth-inhibitory effect by sirolimus was observed at all time points tested (Figure 4A). Indeed, 2 ng/mL of sirolimus suffice to maximally inhibit cell growth. Importantly, high concentrations of extracellular Mg do not rescue the growth-inhibitory effect of sirolimus. We also found that culture for 72 h with sirolimus inhibits rSMC migration (Figure 4B). 3 mM Mg alone reduces migration compared to 1 mM Mg, in accordance with previous reports [8], and does not interfere with the inhibitory effect of sirolimus. 

### 3.5. The Effect of Sirolimus and Mg on the Response of rSMC Growth to NO Donor

We then tested whether sirolimus alters rSMC response to NO, which regulates the degree of contraction of vascular smooth muscle cells by stimulating the production of cyclic GMP (cGMP).

**Figure 5:**
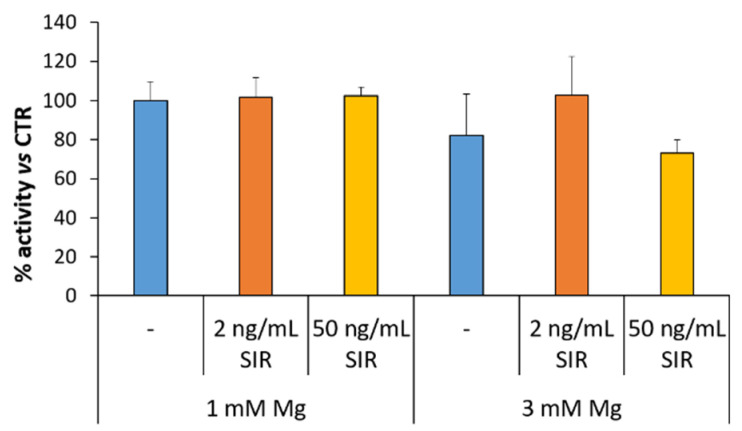
The effects of sirolimus and Mg on the response of rSMC to SNP. The cells were cultured in medium containing 1 or 3 mM Mg with or without sirolimus (2 and 50 ng/mL). After 72 h, the cells were treated with SNP for 30 min. cGMP was measured as described in the methods. Data are expressed as the mean ± standard deviation of three independent assays.

rSMC were cultured in medium containing 1 or 3 mM Mg with or without sirolimus (2 and 50 ng/mL) for 72 h. Then, sodium nitroprusside (SNP 10 µM), a vasodilator which acts as a NO donor, was added for 30 min. Therefore, we measured cGMP as a marker of the sensitivity of rSMC to NO [23]. ELISA shows no alterations in the response to SNP of rSMC cultured in 1 and 3 mM Mg, with or without sirolimus (Figure 5).

## 4. Discussion

Sirolimus-eluting resorbable Mg alloy scaffolds have recently entered clinical practice to treat coronary lesions. The choice of Mg alloy to generate the scaffold is based on the mechanical and biological properties of the metal. From a mechanical point-of-view, Mg provides a radial strength which is adequate to support the vascular wall and offers a high collapse pressure with low elastic recoil [24]. Most of the Mg of the alloy is completely resorbed in about 12 months without side effects [25]. Actually, an increase in Mg concentration in the vessel wall might be beneficial, because of its anti-arrhythmic, anti-thrombotic and anti-inflammatory effects [7,8,9]. Moreover, high extracellular Mg induces quiescence in human coronary SMC, while it stimulates the proliferation of human coronary endothelial cells [18].

Sirolimus, which possesses potent anti-proliferative properties, is frequently used to coat scaffolds. The potential reciprocal interaction between Mg and sirolimus has not been investigated yet. In rabbits, the implantation of Mg-based bioresorbable scaffolds accelerated re-endothelialization and decreased macrophage infiltration in the neointima [2]. Our in vitro results in rCAEC and rSMC might offer some hints to interpret these results. A first issue to consider is that in the in vitro studies performed on endothelial and smooth muscle cells available in the literature, high concentrations of extracellular Mg were used [9,18,26], i.e., 5 mM or more, which is unlikely to be reached even in strict proximity to the scaffold. In addition, in pigs, a Mg-based scaffold increases Mg concentration in the vascular wall up to ~2 mM [18]. This is the reason why we cultured rCAEC and rSMC in a range between 1 and 3 mM Mg. A second point to raise is that our experiments were performed for long times, up to 144 h, while most studies investigated cellular events in the short term.

An interesting finding is that concentrations higher than 1 mM of Mg prevent the inhibitory effect of sirolimus on endothelial proliferation. Moreover, 3 mM Mg with or without sirolimus stimulate migration. These results suggest that re-endothelialization should be granted by a modest increase in extracellular Mg, which counterbalances the inhibitory effects of sirolimus on the endothelium. Turning our attention to endothelial function, we found that high extracellular Mg downregulates VCAM after 72 h of culture, a beneficial event that might reduce the adhesion of leukocytes to the endothelium. However, after 144 h, sirolimus (50 ng/mL) increases the total amounts of VCAM. At the moment, we have no explanation for these results, which raise the possibility of a late sirolimus-induced enhancement of leukocyte adhesion. It would be interesting to investigate VCAM in animal models at various times after the implant of vascular scaffolds. We also measured the amounts of tF, the critical initiator of blood coagulation in acute coronary syndrome, and found no upregulation by sirolimus neither alone nor in combination with Mg. Surprisingly, we found a reduction of tF after 144 h in rCAEC cultured in 3 mM of Mg and treated with sirolimus. In general, these results support the in vivo evidence that Mg-based sirolimus eluting scaffolds are less thrombogenic than other absorbable devices in rabbits [27] and in humans [11].

In rSMC, high extracellular Mg does not interfere with the anti-proliferative and anti-migratory effects of sirolimus. Actually, high Mg potentiates sirolimus in inhibiting rSMC migration. Morever, sirolimus and Mg do not alter rSMC sensitivity to NO donors, thereby suggesting that vasomotion in vivo might be maintained. It should be recalled that Mg might favor vasodilation also by antagonizing Ca [28]. Again, these results should be confirmed in preclinical animal models.

In brief, these in vitro experiments show that 3 mM of extracellular Mg suffice to prevent events associated with neointima formation and in-stent restenosis, contrast endothelial acquisition of pro-thrombotic phenotype and stimulate endothelial growth and migration.

## Data Availability

Data available in a publicly accessible repository. The data presented in this study are openly available in Dataverse at https://dataverse.unimi.it/dataverse/materials (accessed on 14 April 2021).
